# Molecular Interactions and Mechanisms of COVID-19 Inhibition 2.0

**DOI:** 10.3390/ijms25137172

**Published:** 2024-06-29

**Authors:** Francesco Caruso, Miriam Rossi

**Affiliations:** Department of Chemistry, Vassar College, Poughkeepsie, NY 12604, USA; rossi@vassar.edu

Version 2.0 of this Special Issue describes a wide variety of potential targets for COVID-19 and related properties [1]. Redox pathways associated with reactive oxygen and nitrogen species (ROS, RNS) were verified in the sera of COVID-19 patients. Significantly elevated serum levels of ROS and RNS were found in intensive care unit (ICU) patients, compared with non-ICU patients [2]. A peptide that was chemically “stapled” in order to stabilize its alpha-helical structure was able to interfere with virus entry into ACE2-expressing cells [3]. In a specific attempt to find a potential drug, effective against COVID-19, Gramicidin D showed the best in silico interaction profile with PLpro. Gramicidin D was also screened for protease inhibition in vitro, and its inhibitor action was confirmed [4]. Transmembrane protease serine 2 (TMPRSS2) cleaves and proteolytically activates its S protein, which is necessary for viral infection. Schizophyllum commune (SC), which is often found on the rotten wood of trees and has various health benefits, significantly diminished the expression of the ACE2 and TMPRSS2 proteins in vitro and in vivo without cell damage [5]. S-adenosylmethionine analogs were studied as potential inhibitors of methyltransferases enzymes, which are responsible for RNA capping into SARS-CoV-2. Molecular dynamics computational techniques showed the most potent inhibitor with the lowest binding free energy (–58.75 Kcal/mol), which was more powerful than the pan-MTase inhibitor Sinefungin (–39.8 Kcal/mol) [6]. The effects of omega-3 polyunsaturated fatty acids on ACE1 and ACE2 levels were examined in rats and human plasma. Some of these derivatives reduced both ACE1 and ACE2 in the heart, aorta, and kidneys of obese rats, as well as in human endothelial and HEK293 kidney cells. Treatment of HEK293 cells with Docosahexaenoic acid inhibited the entry of the SARS-CoV-2 pseudovirus [7]. Surface plasmon resonance-based technology was used to evaluate the effect of the Delta and Lambda variant mutations on human ACE2 and Bamlanivimab binding. The blocking ability of ACE2 binding by serum antibodies was decreased more by the Lambda than the Delta RBD [8]. Type I and III Interferons are critical cytokines for the antiviral response against epithelium-tropic viruses, as they are effectors of innate immunity and regulators of the development of the adaptive immune response. The virus-mediated evasion of these two interferons was described in three phases [9]. A general overview of the COVID-19 pandemic discussed the hallmarks in its management, from the initial attempts at drug repurposing to the commercialization of Paxlovid, the first orally available COVID-19 drug [10]. The most abundant miRNAs in the control superior temporal lobe neocortex, an anatomical area involved in cognition and targeted by both SARS-CoV-2 invasion and Alzheimer’s disease, were reported for the first time.
